# A Case Report of Yamaguchi Syndrome in a Saudi Male

**DOI:** 10.7759/cureus.52241

**Published:** 2024-01-14

**Authors:** Arwa Almehrij, AlReem Z AlSaleem, Ihab Suliman

**Affiliations:** 1 College of Medicine, King Saud Bin Abdulaziz University for Health Sciences, Riyadh, SAU; 2 Cardiology, King Abdulaziz Medical City, King Abdulaziz Cardiac Center, Ministry of National Guard Health Affairs, Riyadh, SAU

**Keywords:** saudi arabia, saudi, spade like cavity, apical cardiomyopathy, apical hypertrophy cardiomyopathy, apical hypertrophy, apical hypertrophic cardiomyopathy, yamaguchi syndrome, yamaguchi cardiomyopathy

## Abstract

Apical hypertrophic cardiomyopathy, also called Yamaguchi syndrome, is a rare variant of hypertrophic cardiomyopathy. Yamaguchi syndrome is characterized by hypertrophy almost confined to the apical region of the left ventricle rather than the left ventricular septum. A case of 65-year-old Saudi man presented to the ER with angina, and the ECG, echocardiogram, and nuclear study confirmed the diagnosis with Yamaguchi. Reporting this case serves to help physicians broaden their vision in approaching patients with symptoms mimicking acute coronary syndrome.

## Introduction

Apical hypertrophic cardiomyopathy (ApHCM), or Yamaguchi syndrome, is an uncommon subtype of hypertrophic cardiomyopathy. This variant was first described in Japan in 1976 by H. Yamaguchi, and it is characterized by hypertrophy almost confined to the apical region of the left ventricle (LV) rather than the left ventricular septum. Moreover, a spade-like cavity on an echocardiogram is a well-known characteristic of ApHCM [1]. Epidemiologically, it is more common in the Asian population, with a prevalence of 15-25%, compared to the non-Asian population, with 1-10% [2]. The clinical manifestation of Yamaguchi is broad, but the most common presentation is mimicking acute coronary syndrome. Yamaguchi management is mainly symptomatic to control the heart rate, decreasing LV afterload [3]. 

## Case presentation

**Figure 1:**
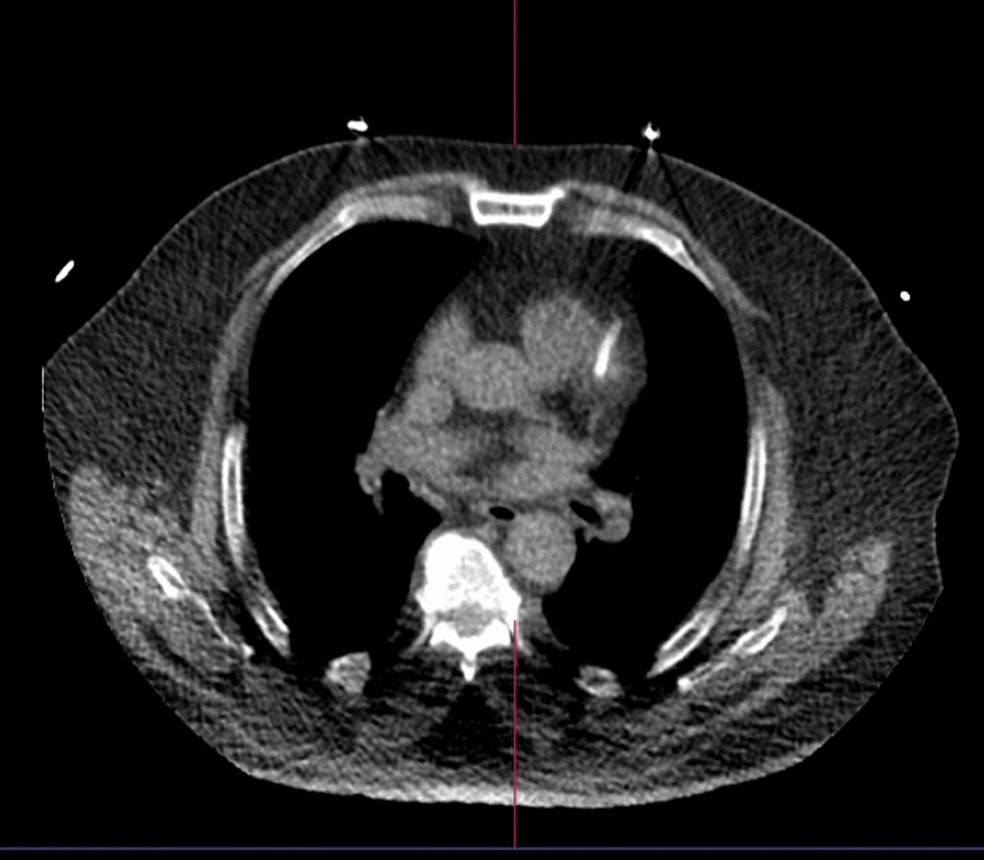
PET scan done at the patient presentation (October 2023) showing LAD stent. LAD: left anterior descending artery.

**Figure 2:**
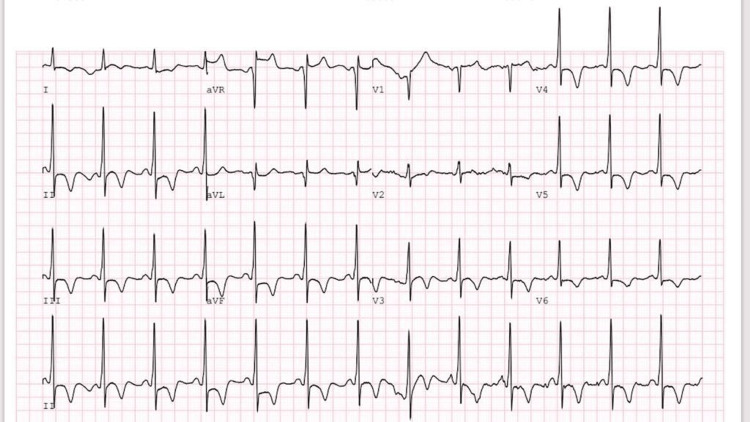
T-wave inversion in almost all leads and ST depression in V4, V5, and V6. This ECG was done at this presentation, and it showed T wave inversion in almost all leads in addition to ST depression in V4, V5, and V6.

A 65-year-old Saudi male known case of hypertension and diabetes mellitus presented to the ER with retrosternal chest pain and tightness associated with dizziness that increased with exertion. His symptoms started after he was stressed with his family. The symptoms lasted for three hours and then subsided. The patient is a smoker and has a negative family history of cardiovascular disease and sudden cardiac death. Furthermore, the patient had anterior non-ST segment elevation myocardial infarction (NSTEMI) two weeks prior to this presentation, and percutaneous coronary intervention (PCI) to the left anterior descending artery (LAD) was done (Figure 1). On examination, the patient had S4 gallop and lower limb pitting edema, while the rest of the examination was unremarkable. In this presentation, 12 leads ECG was obtained and showed T-wave inversion in almost all leads and ST depression in V4, V5, and V6; both were old changes (Figure 2). Troponin I was negative (21.7). The echocardiogram showed a spade-like cavity, which is consistent with ApHCM.

**Figure 3:**
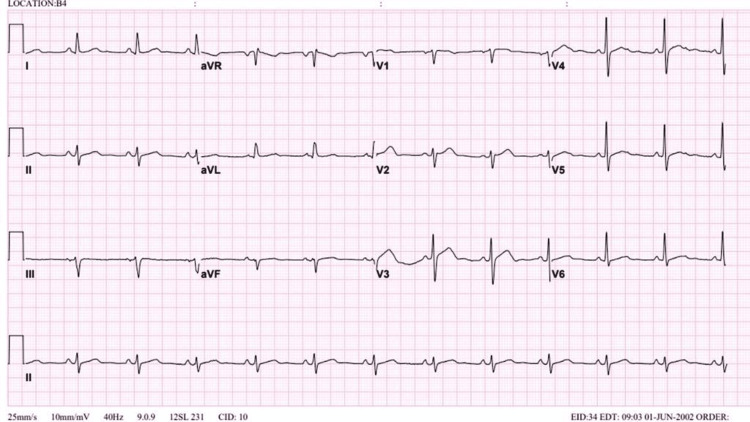
Normal ECG done in 2002.

**Figure 4:**
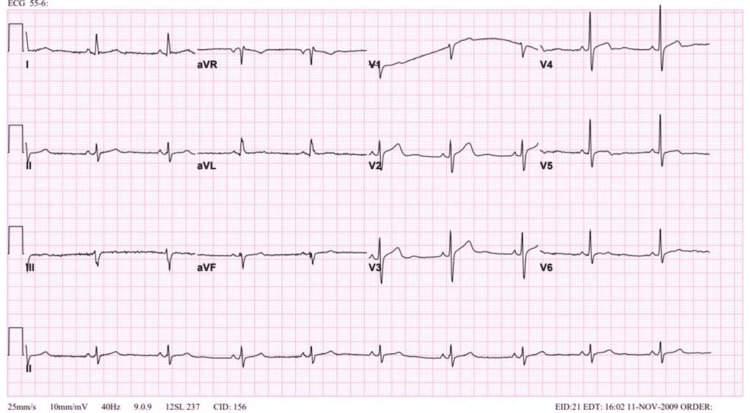
Normal ECG done in 2009.

**Figure 5:**
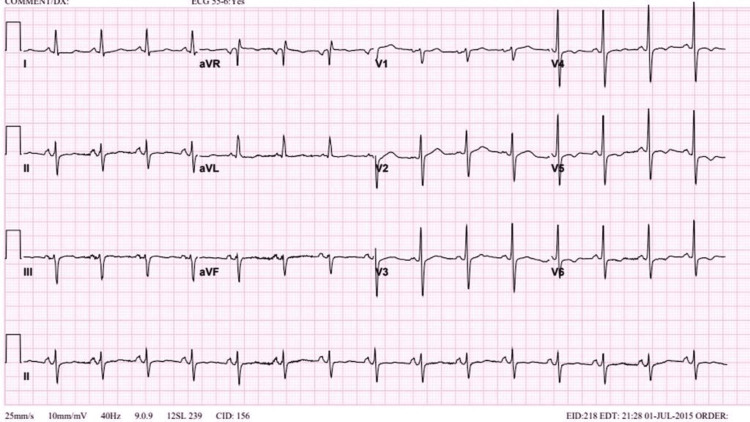
ECG done in 2015: QT segment prolongation and T-wave inversion in V4, V5, and V6.

**Figure 6:**
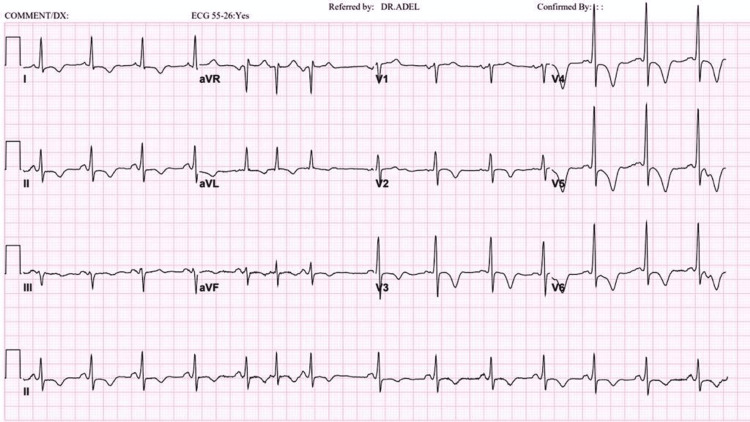
ECG done in 2020: T-wave inversion became deeper compared to the 2015 ECG.

**Figure 7:**
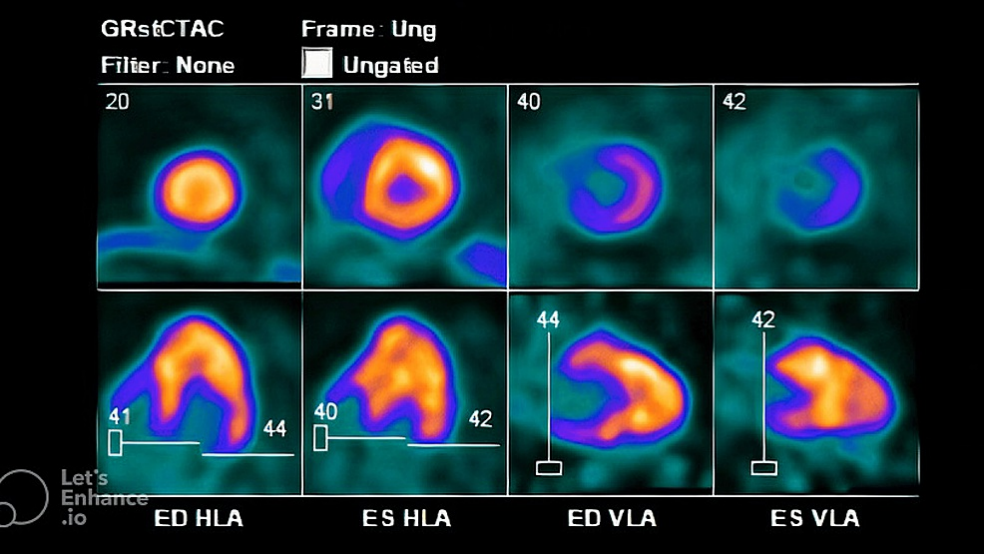
PET scan done at the presentation (in 2023). Rest PET test showing spade-like cavity.

**Figure 8:**
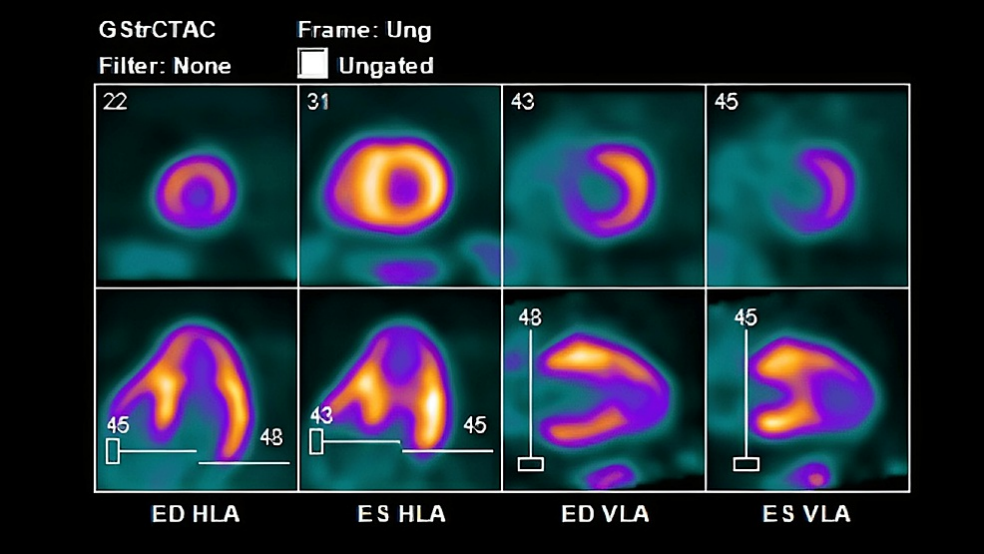
PET scan done at the presentation (in 2023). Stress PET test showing wall defect in the apex of the left ventricle, reflecting LAD ischemia. LAD: left anterior descending artery.

**Figure 9:**
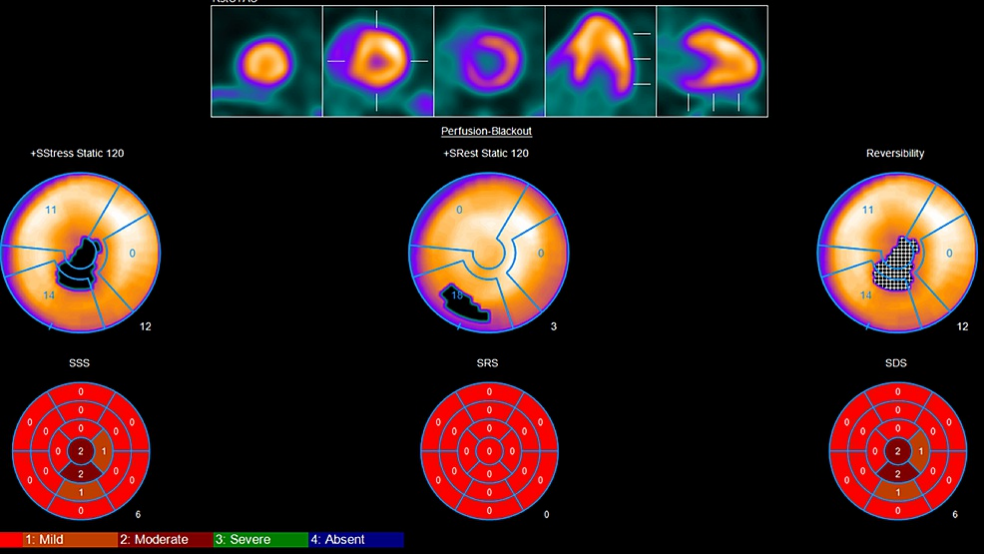
PET scan done at the presentation (in 2023). Showing perfusion defect, suggestive moderate reversible LAD ischemia. LAD: left anterior descending artery.

When reviewing the patient’s previous ECG, we found that in the 2002 and 2009 ECGs (Figures 3, 4), at that time, the patient was 42 and 49 years old, respectively, and no changes were noted. The beginning of the changes was noted in an ECG done in 2015 at the age of 55, which was QT segment prolongation and T-wave inversion in V4, V5, and V6 (Figure 5). Furthermore, the patient presented to the hospital in October 2020 at the age of 60 with chest pain and coughing. An echocardiogram was done, but due to the coughing, the pictures were poor. An ECG was obtained at that time, and the T-wave inversion became deeper compared to the 2015 ECG (Figure 6). During this presentation, a PET scan was done and showed a clear spade-like cavity (Figure 7). In addition, during stress testing, a wall defect was noted in the left ventricular apex. Resting PET confirmed the reversibility of LAD ischemia (Figures 8, 9). An MRI was ordered to complete the workup, but the patient is claustrophobic, which led to the cancelation of the MRI.

The patient was managed medically. He was on heparin, metoprolol (12.5 mg BID), valsartan 80 mg once daily, rosuvastatin 40 mg, amlodipine 10 mg once daily, and indapamide 1.5 mg once daily. Heparin was discontinued after acute coronary syndrome was rolled out. The rest of the medications continued as the patient had ApHCM with HF symptoms. The patient had a good response to the medical therapy and was discharged on the same medications. Follow-up sessions with the patient were reassuring, and no modifications to the regimen were needed.

## Discussion

Retrosternal chest pain and tightness were associated with dizziness and increased with exertion. His symptoms started after having stress with his family. The symptoms lasted for three hours and then subsided. The patient is a smoker and has a negative family history of cardiovascular disease and sudden cardiac death. Furthermore, the patient had anterior NSTEMI two weeks prior to this presentation, and PCI to LAD was done (Figure 1). On examination, the patient had S4 gallop and lower limb pitting edema, while the rest of the examination was unremarkable. In this presentation, 12 leads ECG was obtained and showed T-wave inversion in almost all leads and ST depression in V4, V5, and V6; both were old changes (Figure 2). Troponin I was negative (21.7). The echocardiogram showed a spade-like cavity.

We reported a case of a 65-year-old Saudi male who presented to the ER with retrosternal chest pain and tightness, and the physical examination revealed S4 gallop and lower limb pitting edema. In addition, an ECG and PET scan were done, which confirmed the diagnosis of ApHCM. Dr. Yamaguchi first described ApHCM in Japan in 1976. Yamaguchi syndrome is an autosomal dominant disease and has a male predominance with a male-to-female ratio of approximately 1.6 to 2.8:1 [2]. In addition, the predominant gene mutations occur in "myosin-binding protein C (MYBPC3)" and "myosin heavy chain (MYH7)" [4].

In terms of clinical presentation, ApHCM mostly presents with angina, atypical chest pain, dyspnea, palpitation, syncope, or presyncope [5]. On examination, audible and sometimes palpable S4 is appreciated, reflecting an impairment of LV diastole. Up to 50% of the cases have an impairment to the mitral valve; therefore, a mitral regurgitation murmur can be present as well. The main workups done for patients presenting with chest pain mimicking acute coronary syndrome are ECG, troponin I, MRI, and nuclear scan [6]. The classical ECG findings are deep negative T waves (>10 mm in amplitude) in precordial leads in addition to LVH findings. The spade-like cavity in an echocardiogram is pathognomonic for ApHCM [1].

In terms of management, symptomatic ApHCM is managed medically with a target to reduce the heart rate and LV afterload by using beta-blockers, calcium channel blockers, and angiotensin-converting enzyme inhibitors [3]. Cardiac transplant and apical myomectomy could be offered for patients with severe heart failure symptoms refractory to the medical management [7]. Yamaguchi syndrome has a benign course with annual cardiovascular mortality of 0.1% and overall survival of 95%. A study conducted at Toronto General Hospital found that the probability of survival without morbidity related to ApHMC was 74% in 15 years. Moreover, several complications were reported, with atrial fibrillation being the most common (12%), followed by myocardial infarction (10%) [8].

## Conclusions

Yamaguchi syndrome is traditionally thought to be seen in Asian populations only. Throughout the years, cases have been reported from all over the world. This case report highlights that it can be seen in the Saudi Arabian population as well. Moreover, this case report highlights the importance of following up with patients with recurrent acute coronary syndrome-like presentations. In addition, screening patients' family members is recommended.
